# Supplementary material for: Visual evoked feedforward–feedback traveling waves organize neural activity across the cortical hierarchy in mice
Source: Nat Commun. 2022 Aug 13;13:4754. doi: 10.1038/s41467-022-32378-x (PMC9376099; doi:10.1038/s41467-022-32378-x)
Supplement: Supplementary file 1 — Supplementary Information [file 41467_2022_32378_MOESM1_ESM.pdf]

SUPPLEMENTARY FIGURES

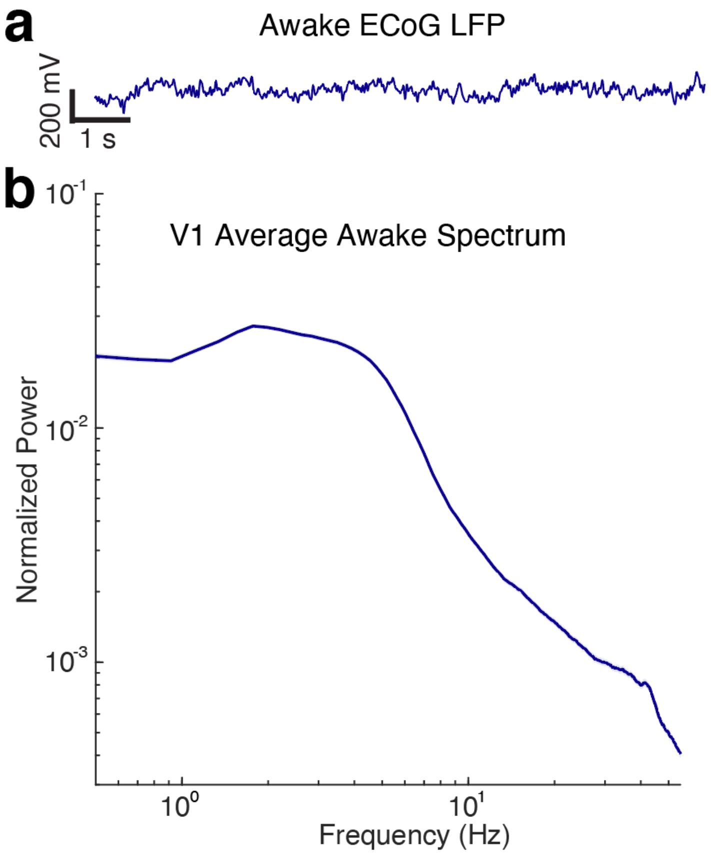

Supplementary Figure 1: Awake LFP and Power spectrum

a. Spontaneous LFP recorded over V1 in a representative awake mouse. Note the dominance of high frequency, low amplitude activity
b. Power spectrum of V1 LFP averaged over animals.

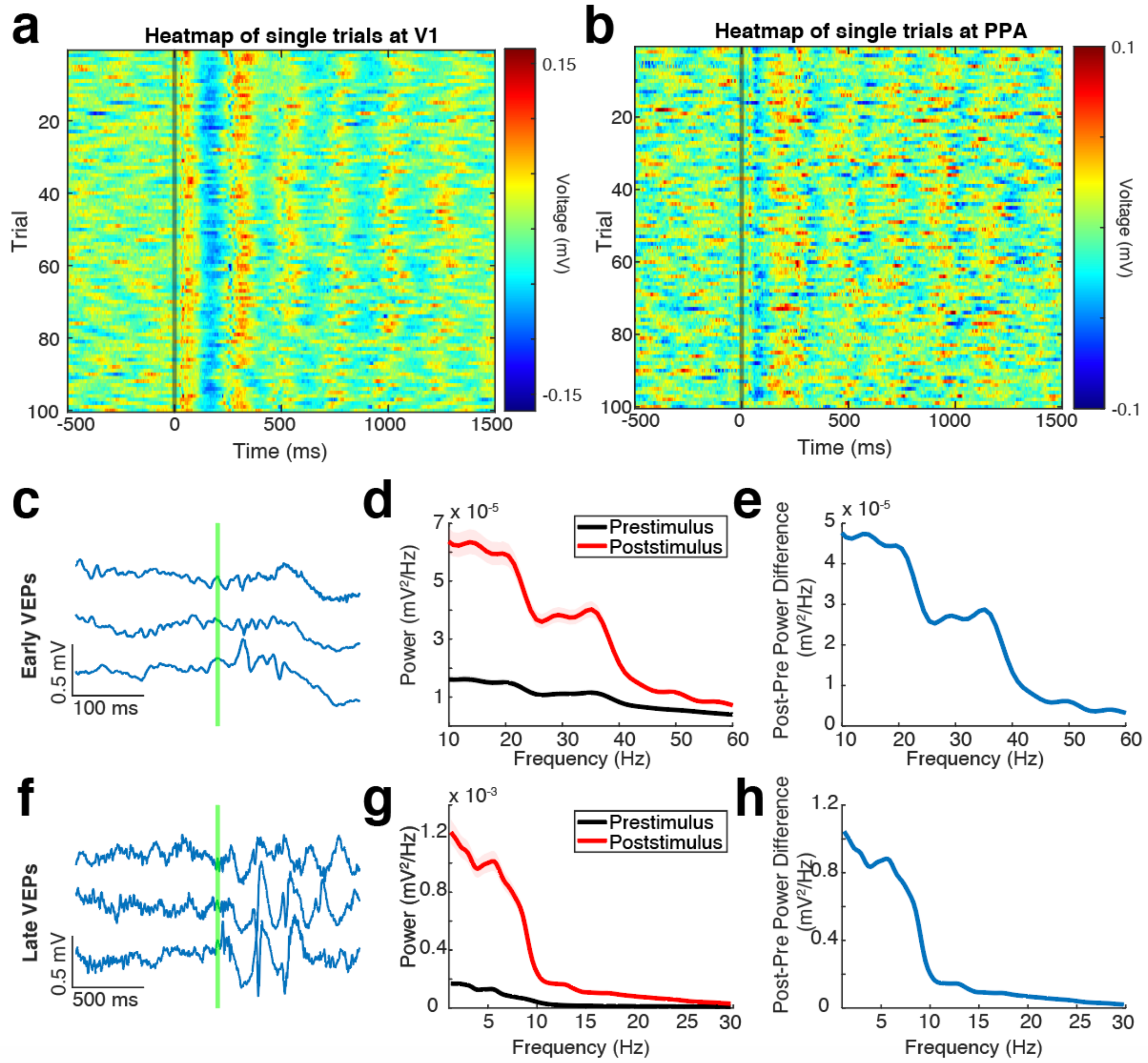

Supplementary Figure 2: Single trials contain fast 30-50 Hz and slow 3-6 Hz oscillations

a. Single trials of VEPs at an electrode over V1, with trial number on the x axis, time on the y axis, and voltage as color in a representative mouse. The gray vertical line depicts the flash onset.
b. Same as A for an electrode over PPA.
c. Three single trials of VEPs (blue traces) over V1 during the baseline and early post stimulus period. The green vertical line denotes visual stimulus onset (LED flash).
d. Across-trial average power spectrum of 150 ms of pre-stimulus activity (black) and 150 ms of post-stimulus activity (red), with 95% Jacknife confidence intervals (shading) display a peak in post-stimulus activity at 30-40 Hz.
e. Difference of average of 150 ms of pre-stimulus and 150 ms of post-stimulus power spectrum similarly displays a peak in post-stimulus activity at 30-40 Hz.

f. Three single trials of VEPs (blue traces) over V1 during the baseline and late post stimulus period. The green vertical line denotes visual stimulus onset.
g. Across-trial average power spectrum of 1 s pre-stimulus activity (black) and 1 s of post-stimulus activity (red), with 95% confidence intervals (shading) display a peak in post-stimulus activity at 4-6 Hz.
h. Difference of average of 1 s pre-stimulus and 1 s of post-stimulus power spectrum similarly displays a peak in post-stimulus activity at 4-6 Hz.

* All data shown in this figure comes from a representative mouse

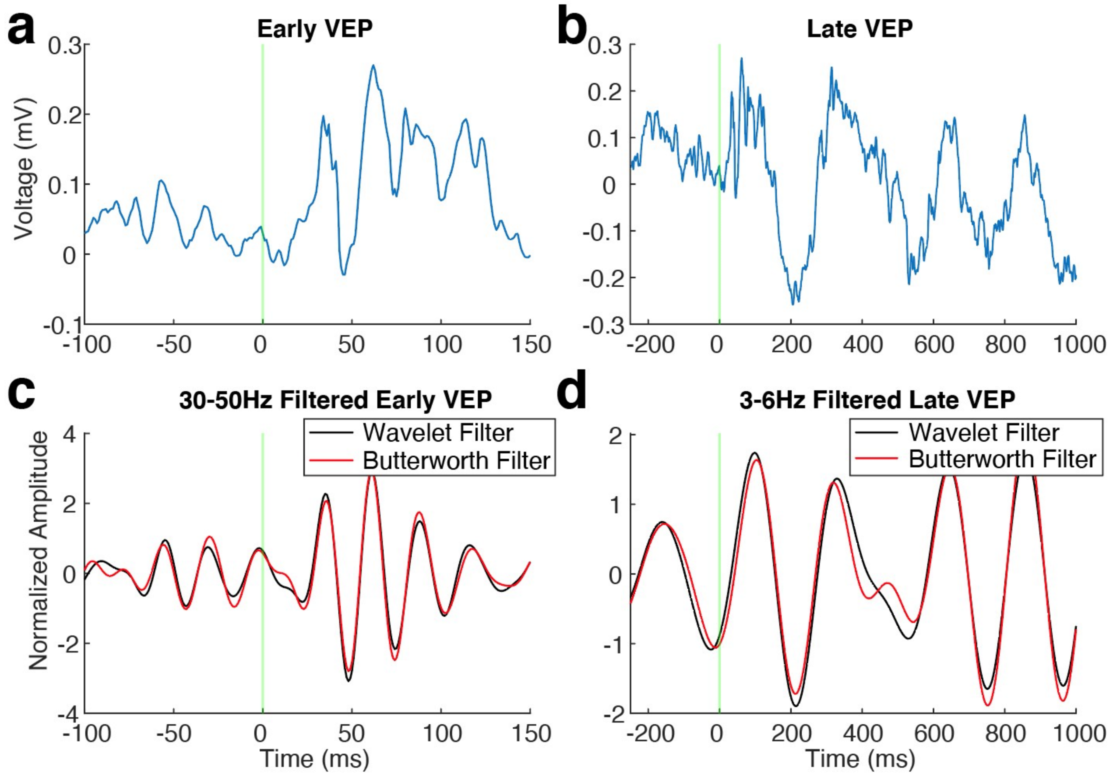

Supplementary Figure 3: Single trials filtered with wavelets and Butterworth filters

a. Example of an early VEP with 100 ms of baseline and 150 ms of post stimulus raw LFP from a representative mouse over V1
b. Example trace of a late VEP with 200 ms of baseline and 1000 ms of post stimulus raw LFP from a representative mouse over V1
c. Early VEP in (a) filtered at 30-50 Hz using a series of wavelets (black) and a Butterworth filter (red)
d. Late VEP in (b) filtered at 3-6 Hz using a series of wavelets (black) and a Butterworth filter (red)

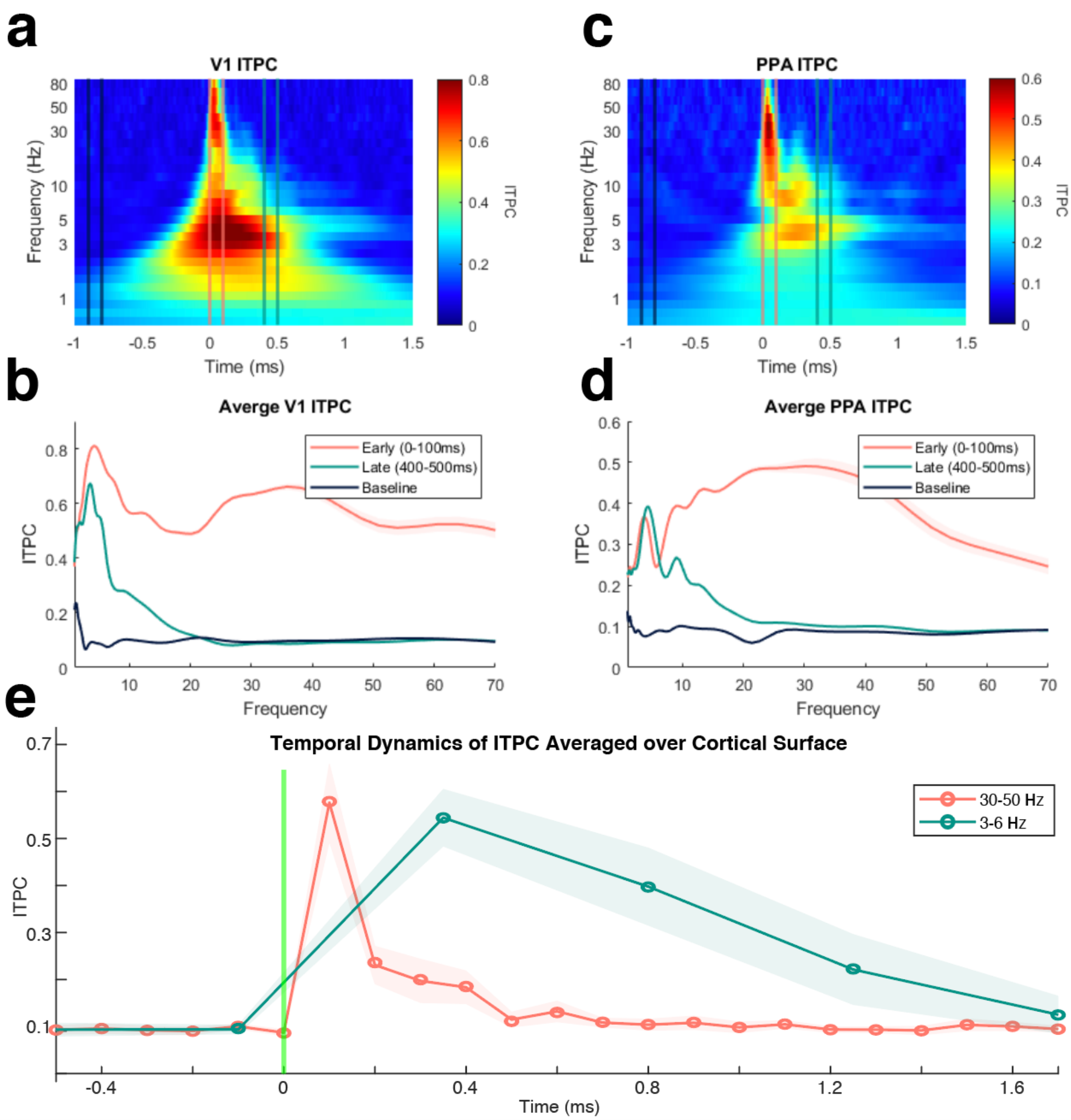

Supplementary Figure 4: 30-50 Hz dominates ITPC in the early post-stimulus period, while 3-6Hz ITPC dominates ITPC later after stimulus onset.

a. Inter-trial phase coherence (ITPC) computed using wavelets at V1 and averaged over single trials and animals (0 ms marks stimulus onset).
b. Time slices through the wavelet coherogram at V1 shown in a. Colors of the traces correspond to time segments shown in a. The thick line shows the average. Shaded areas show 95% confidence intervals.
c. Inter-trial phase coherence (ITPC) computed using wavelets at PPA and averaged over single trials and animals (0 ms marks stimulus onset).

d. Time slices through the wavelet coherogram at PPA shown in c. Colors of the traces correspond to time segments shown in a. The thick line shows the average. Shaded areas show 95% confidence intervals.
e. Average 30-50 Hz (orange) and 3-6 Hz (teal) ITPC over the grid calculated using multitaper spectral analysis in nonoverlapping windows. Center of each window is shown by circles. Shading represents the 95% confidence intervals of the mean at each time point. This highlights the temporal dynamics of the fast and slow oscillation coherence and eliminates the artifactual increase in 3-6Hz coherence before the stimulus.

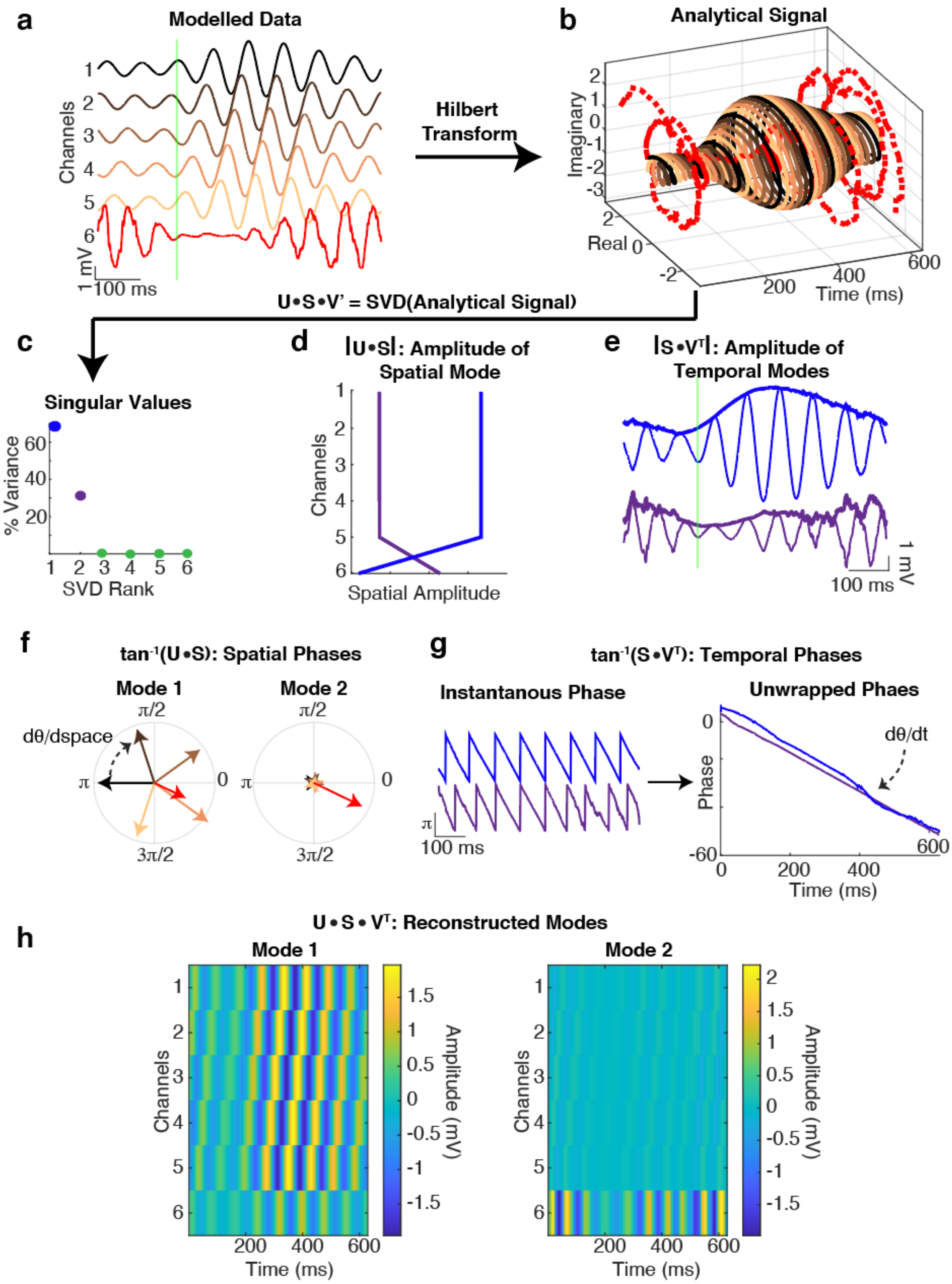
a
Modelled Data
Channels
1 mV
100 ms
Hilbert Transform
b
Analytical Signal
Imaginary
Real
Time (ms)
U•S•V' = SVD(Analytical Signal)
c
Singular Values
% Variance
SVD Rank
d
|U•S|: Amplitude of Spatial Mode
Channels
Spatial Amplitude
e
|S•V^T|: Amplitude of Temporal Modes
1 mV
100 ms
f
tan^-1(U•S): Spatial Phases
Mode 1
Mode 2
dθ/dspace
g
tan^-1(S•V^T): Temporal Phases
Instantanous Phase
Unwrapped Phaes
dθ/dt
Phase
Time (ms)
h
U • S • V^T: Reconstructed Modes
Mode 1
Mode 2
Channels
Amplitude (mV)
Time (ms)

Supplementary Figure 5: Singular value decomposition (SVD) of the analytical signal data identifies coherent spatiotemporal activity modes.

a. Modelled oscillatory activity for 6 electrodes. The signals in the first five electrodes record a traveling wave that propagates from electrode 5 to 1 (black through orange) The sixth electrode (red), is constructed to be independent from others. The green vertical line denotes the stimulus.
b. Analytical (complex valued) signal is obtained using the Hilbert transform from data in A. In the complex plane, the travelling wave appears as phase shifted copies of the same signal and the divergent activity of the sixth electrode is distinct.
c. Singular value decomposition (SVD) can be used to parse the analytical signal into mutually orthogonal modes. Real valued diagonal matrix S encodes the relative contribution of each mode to the overall activity pattern. Complex-valued U and V matrices encode the spatial and temporal characteristics of each mode respectively. SVD identifies only two modes with nonzero singular values, corresponding to the two oscillatory patterns within the signal.
d. The columns of matrix $|\boldsymbol{U}*\boldsymbol{S}|$ encode the spatial amplitude of each mode, and measures how much each electrode contributes to each of the temporal modes. SVD correctly identifies that the first 5 electrodes contribute to the first mode (the traveling wave) equally, whereas mode 2 exclusively involves electrode 6.
e. The temporal amplitudes, computed as $|\boldsymbol{S}*\boldsymbol{V}^{\boldsymbol{T}}|$, reveal the envelope of each mode. Visually responsive modes were defined as modes that increase in temporal amplitude after the stimulus. In this example, only the first mode is visually responsive.
f. The spatial phases are calculated as arctangent of $\boldsymbol{U}$ scaled by the corresponding singular value. The spatial frequency is computed from the phase gradient ($^{d\theta}/_{ds} = F_s$). Here, the phase map of the first mode illustrates that the first five electrodes contribute (black through orange) equally and have a constant phase offset from one another, whereas the sixth electrode (red) has a smaller magnitude. In the second mode, only the sixth electrode has a large magnitude.
g. The temporal phases are calculated as arctan of $\boldsymbol{V}^{\boldsymbol{T}}$. The time derivative of this phase ($^{d\theta}/_{dt} = F_t$) defines the temporal frequency of the mode and is approximated by measuring the slope of the unwrapped temporal phase. The propagation velocity of the mode is then computed as $^{F_t}/_{F_s}$.
h. The activity corresponding to the i-th spatiotemporal mode can be reconstructed as $U_{:,i} * S_{i,i} * V_{i,:}^{T}$. Here, the first mode shows a traveling wave in the first 5 electrodes. The independent spatiotemporal mode in the sixth electrode is present in the second mode.

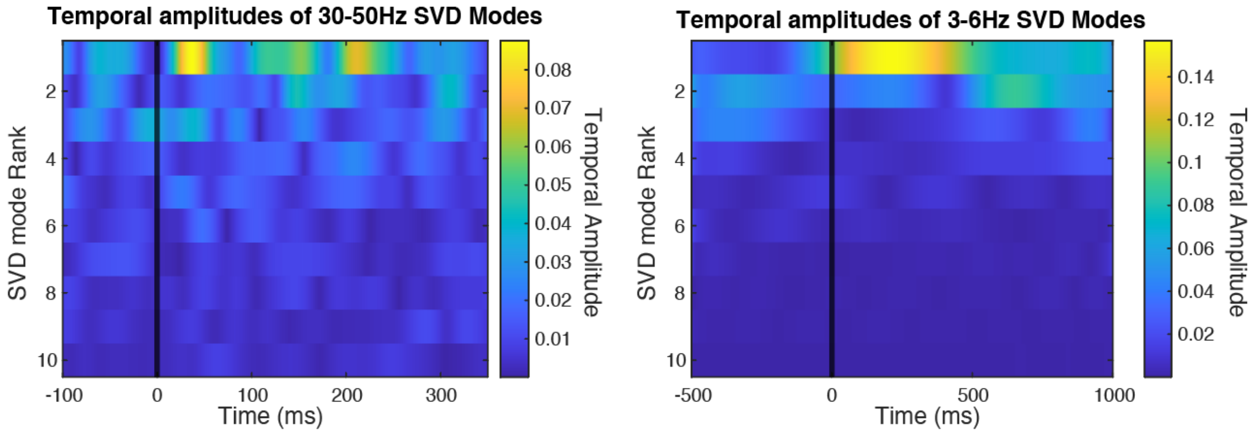

Supplementary Figure 6: The first mode extracted from SVD typically has the highest post-stimulus temporal amplitude

Temporal amplitude of each of the first 10 SVD modes of a single trial filtered at 30-50Hz (right) or 3-6Hz (left), by time in ms. The stimulus occurs at the black line. Note in both frequency bands, the first mode has the largest increase in amplitude following the stimulus and is therefore defined as the most responsive visual mode.

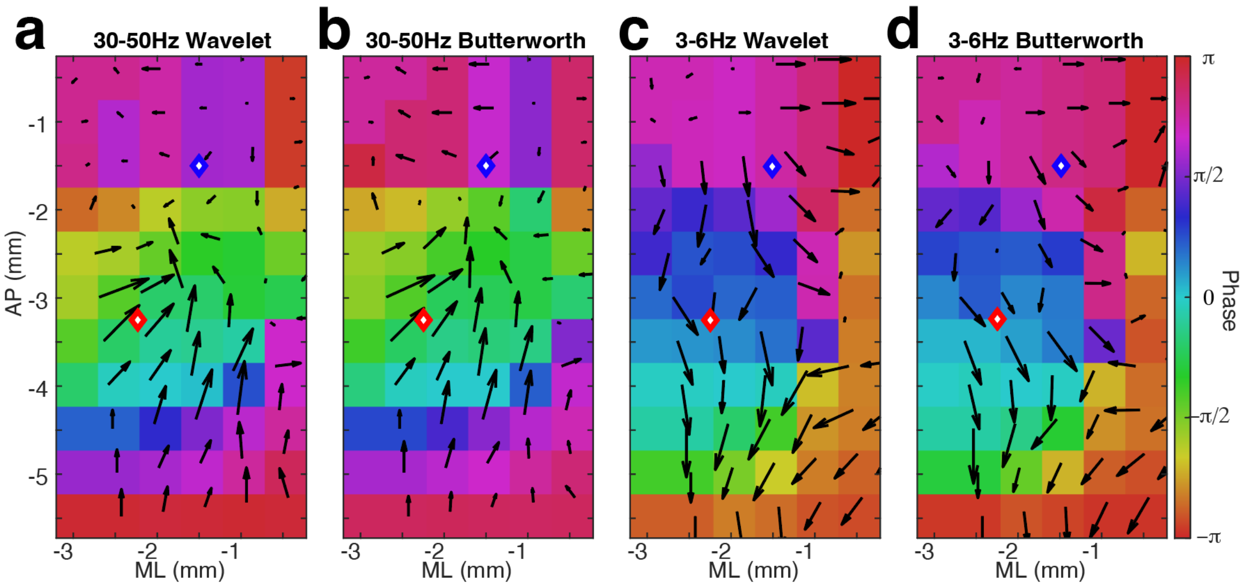

Supplementary Figure 7: SVD phase plots and spatial gradients are robust to filtering techniques

a. At each stereotaxic location, the average phase offset of the 30-50Hz spatial mode relative to V1 (the red diamond) elicited by the LED stimulus is plotted in color. The location of PPA is denoted by the blue diamond. Spatial phase gradient is depicted by black arrows. The direction of the arrows shows the direction of spatial phase gradient averaged over trials in a single representative mouse. The magnitude of the arrows corresponds to the consistency of the angle of the spatial phase gradient over trials and animals.
b. Same plot as in A, but for 30-50 Hz waves data extracted using a Butterworth filter (Methods).
c. Same plots as in A but for waves identified from 3-6 Hz data extracted using wavelets
d. Same plots as in C but for waves identified from 3-6 Hz data extracted using a Butterworth filter

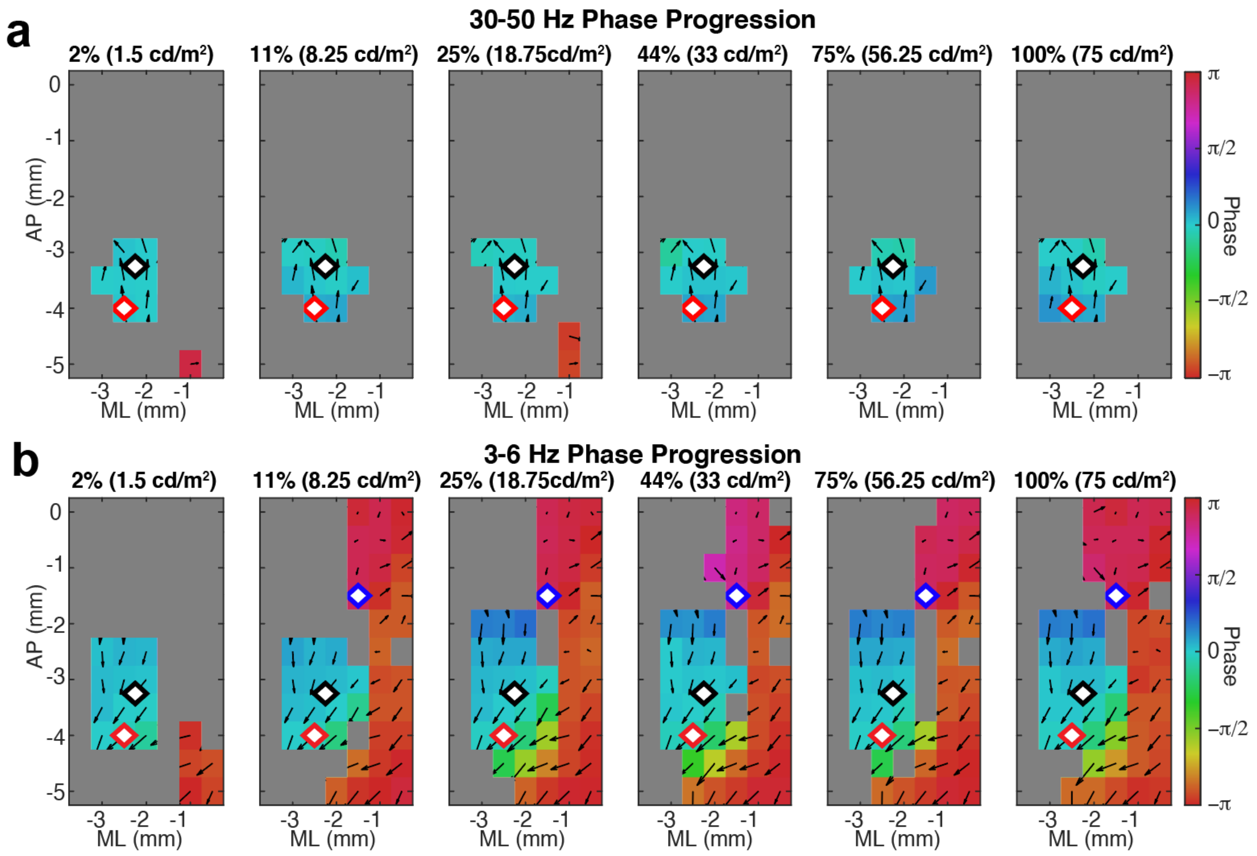

Supplementary Figure 8: The consistency of the wave propagation pattern depends on stimulus intensity

a. At each stereotaxic location, the average (over mice) phase offset of the 30-50Hz spatial mode relative to V1 (the black diamond) is plotted in color for each screen luminance, listed as a percent of maximum screen luminance. The red diamond is a different location in V1. Spatial phase gradient is depicted by black arrows. The direction of the arrows shows the direction of spatial phase gradient over trials and mice. The magnitude of the arrows corresponds to the consistency of the angle of the spatial phase gradient over trials and animals. Locations that are grayed out did not meet Bonferroni corrected statistical significance (p-value < 0.0006, Rayleigh test) across animals.

b. Same plots as in A but for waves identified from 3-6Hz filtered data. The blue diamond denotes the location of the PPA.

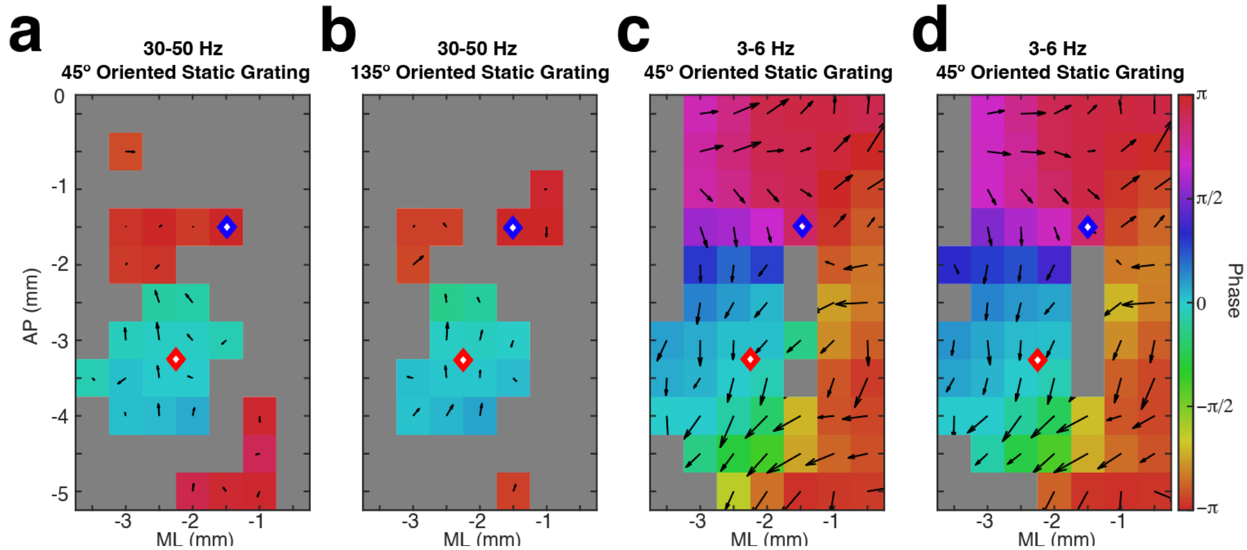

Supplementary Figure 9: Static gratings at different orientations also elicit fast feedforward waves within V1, and slower feedback waves across the cortical surface

a. At each stereotaxic location, the average (over mice) phase offset of the 30-50Hz spatial mode relative to V1 (the red diamond) is plotted in color for static gratings oriented at 45°. The location of PPA is denoted by the blue diamond. Spatial phase gradient is depicted by black arrows. The direction of the arrows shows the direction of spatial phase gradient over trials and mice. The magnitude of the arrows corresponds to the consistency of the angle of the spatial phase gradient over trials and animals. Locations that are grayed out did not meet Bonferroni corrected statistical significance (p-value < 0.0006, Rayleigh test) across animals.
b. Same plot as in A, but for 30-50 Hz waves elicited from static gratings oriented at 135°
c. Same plots as in A but for waves identified from 3-6 Hz filtered data elicited from static gratings oriented at 45°.
d. Same plots as in A but for waves identified from 3-6 Hz filtered data elicited from static gratings oriented at 135°.

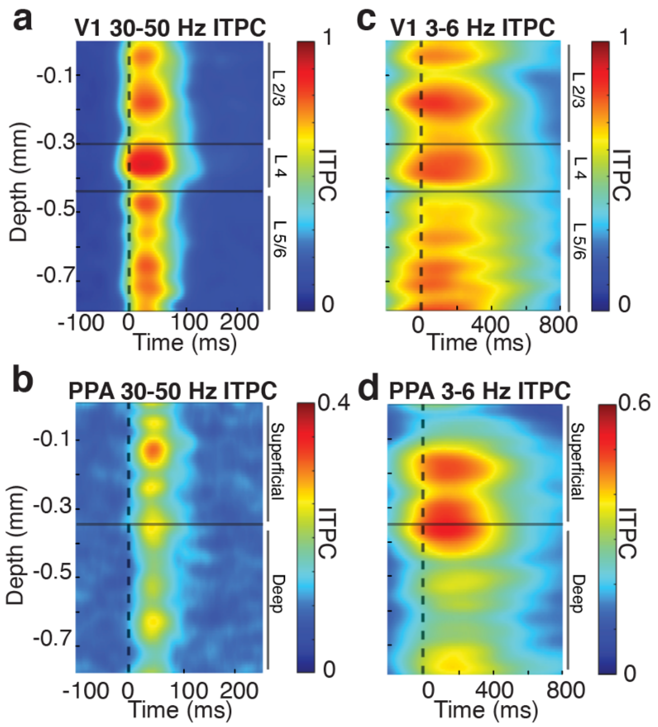

Supplementary Figure 10: Superficial cortical layers of V1 and PPA contain high ITPC

a. ITPC of CSD at 30-50Hz as a function of time and depth in V1, averaged over animals. The horizontal lines indicate the supra-, granular, and infragranular layers.
b. Same plots as in A but for PPA. Note that high frequency ITPC is most prominent in superficial cortical layers.
c. ITPC of CSD at 3-6Hz as a function of time and depth in V1, averaged over animals. The horizontal lines indicate the superficial and deep layers.
d. Same plots as C but for PPA. In contrast to V1, ITPC is most dominant in the superficial layers.

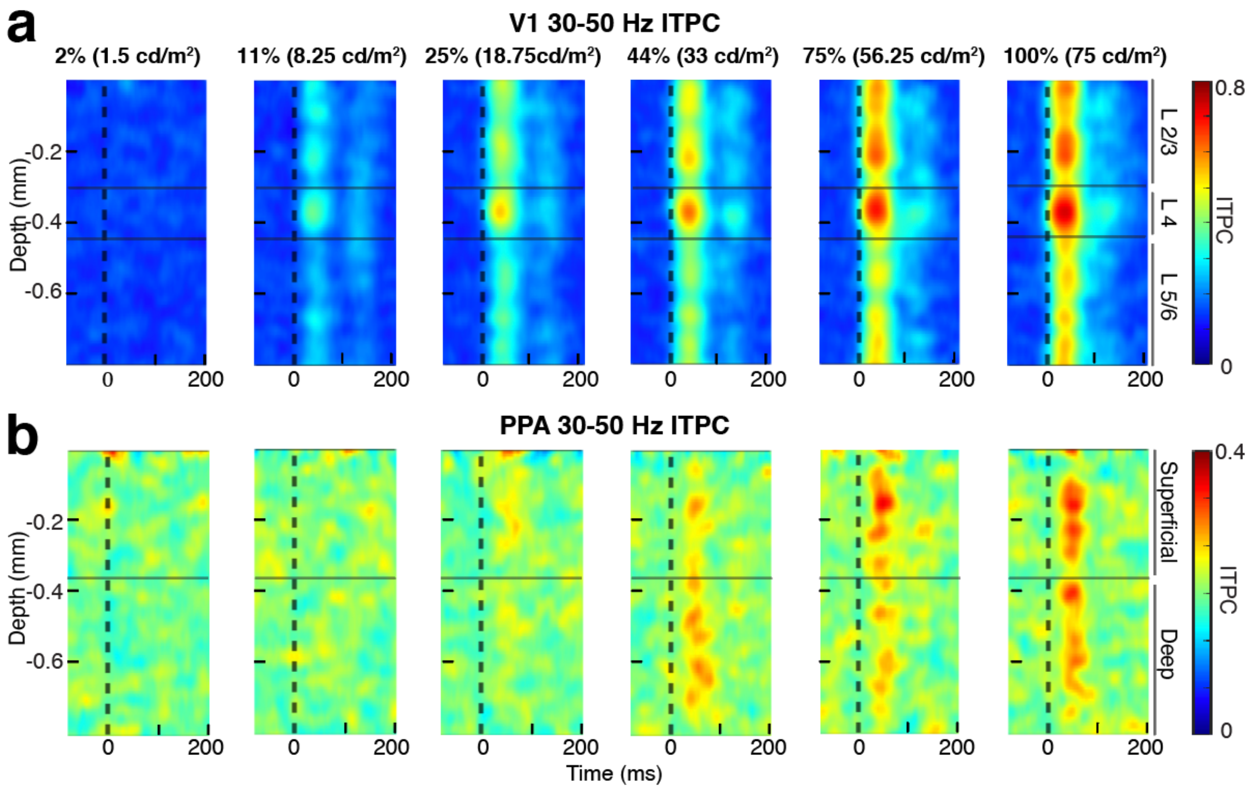

Supplementary Figure 11: 30-50 Hz intertrial phase coherence is present in laminar data for lower intensity stimuli (full screen flash)

a. ITPC of CSD at 30-50Hz as a function of time and depth in V1 for each intensity screen flash, averaged over animals. The horizontal lines indicate the supra-, granular, and infragranular layers. The dashed vertical line corresponds to stimulus onset.
b. Same plots as in A but for PPA.

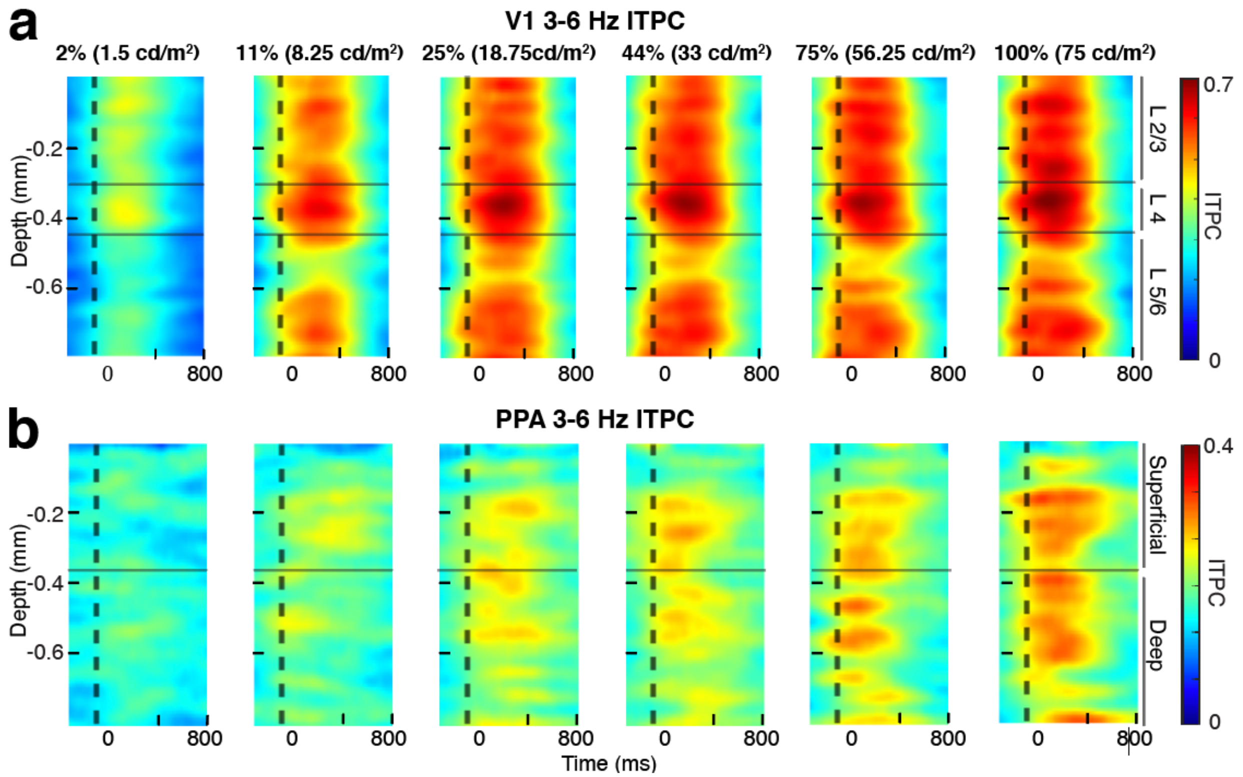

Supplementary Figure 12: 3-6 Hz intertrial phase coherence is present in laminar data for lower intensity stimuli (full screen flash)

a. ITPC of CSD at 3-6 Hz as a function of time and depth in V1 for each intensity screen flash, averaged over animals. The horizontal lines indicate the supra-, granular, and infragranular layers. The dashed vertical line corresponds to stimulus onset.

b. Same plots as in A but for PPA.

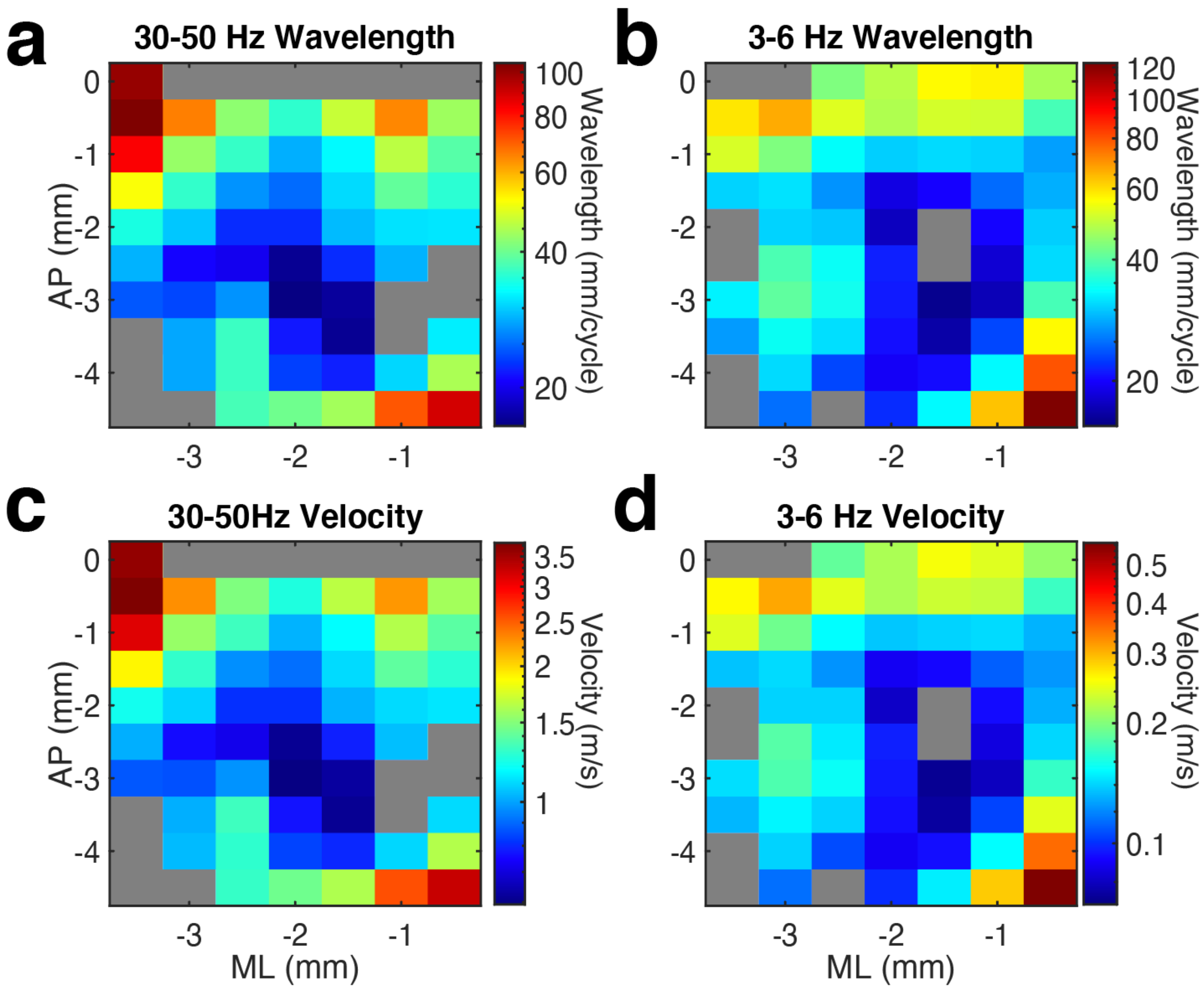

Supplementary Figure 13: Spatial wavelength and wave velocity are not uniform over space

a. Spatial wavelength of fast 30-50Hz most visually responsive SVD modes.
b. Spatial wavelength of fast 30-50Hz most visually responsive SVD modes.
c. Velocity of fast 30-50Hz most visually responsive SVD modes.
d. Velocity of fast 30-50Hz most visually responsive SVD modes.

*Note that in all plots, the color axis is in log scale and locations that are grayed out did not meet Bonferroni corrected statistical significance (p-value < 0.0006, one-sided Stouffer's test) across animals.

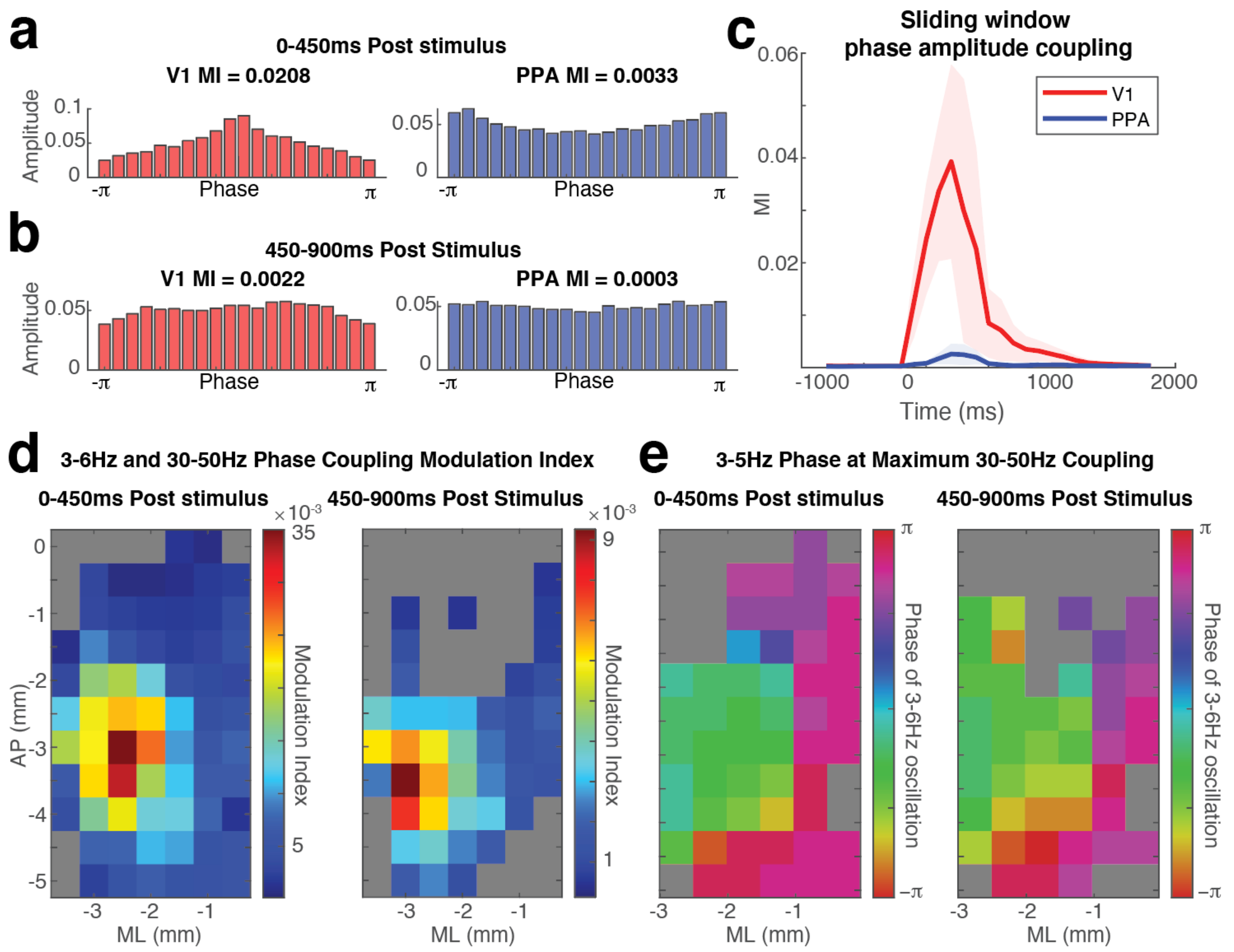

Supplementary Figure 14: Phase amplitude coupling is strongest within the first 450 ms

a. Amplitude of 30-50 Hz oscillations as a function of phase of the low frequency oscillation, averaged over trials in V1 (red, left) and PPA (blue, right) during the first 450 ms after stimulus onset. The deviation of this distribution from a uniform distribution is summarized in the modulation index (MI) (p-value <0.0001, one-sided student's t-test, compared to time shuffled surrogates)
b. Similar to A but for 450-900 ms after stimulus onset. Note that the phase amplitude histograms are more uniform and consequently the MIs at V1 and PPA are lower than those observed during the first 450 ms of post-stimulus activity (p-value <0.05 for V1, one-sided student's t-test, compared to time shuffled surrogates).
c. MI calculated over a 200 ms sliding window averaged over trials and animals at V1 and PPA. Shading represents the 95% confidence intervals of the mean at each time point.
d. Modulation indices averaged over all mice and plotted in color at each stereotaxic location for the first 450 ms (left) and 450-900 ms (right) of post-stimulus activity. Locations that are grayed out did not meet Bonferroni corrected statistical significance (p-value < 0.0006, Rayleigh test) compared to time shifted surrogate data. MI peaks near V1 but remains statistically significant over much of the cortical surface.

e. The phase of the slow 3-6Hz oscillation at which the fast 30-50Hz oscillation reaches maximum amplitude as shown for a representative mouse at each stereotaxic location during the first 450 ms (left) and 450-900 ms (right) of post-stimulus activity. Grayed out locations did not meet statistical significance compared to time shifted surrogates.

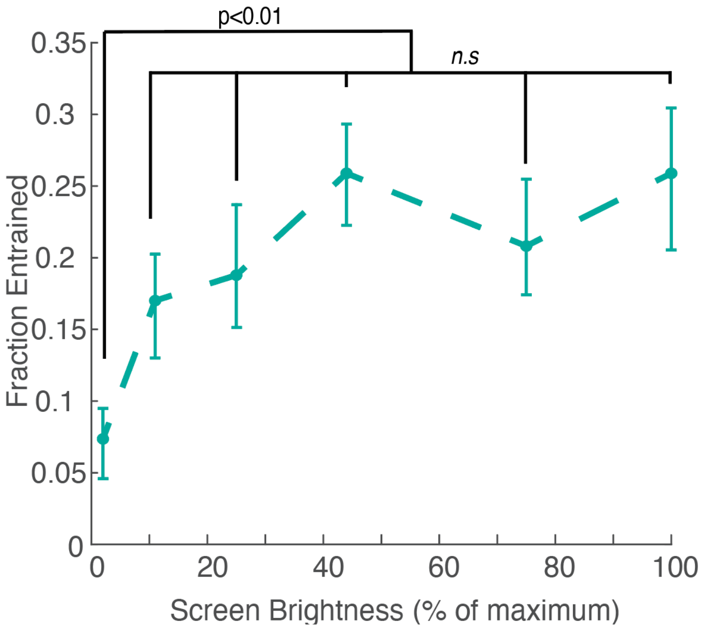

Supplementary Figure 15: The proportions of cells entrained by the slow wave increases with stimulus intensity for full screen flashes.

The proportion of neurons in V1 and PPA (394 total cells) that become entrained to the slow wave (y-axis) increases from 2% to 11% screen flash intensity, but remains relatively constant for higher intensity stimuli. Error bars show bootstrap 95% confidence intervals, comparison statistics calculated using a one way Tukey test. Entrainment at a screen brightness of 2% is statistically lower compared to all other screen luminance conditions (maximum p-value = 0.001, between 2% and 11%). Entrainment at 11% screen brightness is lower than entrainment at 44% brightness (p-value = 0.028) and lower than entrainment at 100% screen brightness (p-value = 0.028). All other comparisons are not statistically significant at an $\alpha = 0.05$)

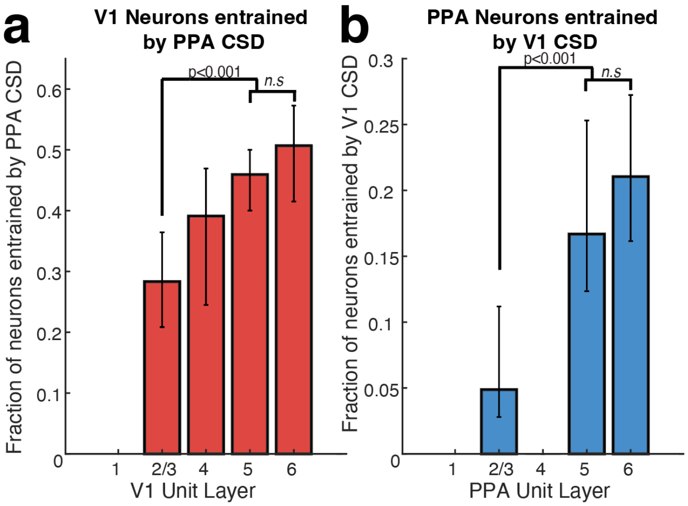

Supplementary Figure 16: Cross Site Spike Field Coherence

a. Phase of the slow oscillations PPA was extracted from the CSD at 6 cortical depths corresponding to cortical layers. Layer assignment is approximate based on cortical depth. SFC was computed for each electrode in V1. The fraction of neurons reaching statistical significance (z-score≥4) threshold found in each layer are plotted as the bars. Error bars show bootstrap 95% confidence intervals, comparison statistics calculated using a one-way Kruskal Wallis (p-value = 2.37×10^{-101} for PPA neurons entrained by V1 cells) and pairwise comparisons were done using two-way Student's t-tests. Layer 2/3 PPA neurons were less entrained by V1 cells than Layer 5 (p-value = 8.31×10^{-4}) and Layer 6 PPA neurons (p-value = 7.72×10^{-8}).

b. Phase of the slow oscillations V1 was extracted from the CSD at 6 cortical depths corresponding to cortical layers. Layer assignment is for V1 layer assignment made on the basis of the CSD. SFC was computed for each electrode in PPA. The fraction of neurons reaching statistical significance (z-score≥4) threshold found in each layer are plotted as the bars. Error bars show bootstrap 95% confidence intervals, comparison statistics calculated using a one-way Kruskal Wallis (p-value = 1.98×10^{-93} for V1 neurons entrained by PPA cells). Layer 2/3 V1 neurons were less entrained by PPA cells than Layer 5 (p-value = 8.04×10^{-8}) and Layer 6 neurons (p-value = 3.82×10^{-5}), but were as entrained by PPA neurons at Layer 4 V1 cells (p-value > 0.05), student's t-test.
