# Supplementary material for: Visual evoked feedforward–feedback traveling waves organize neural activity across the cortical hierarchy in mice
Source: Nat Commun. 2022 Aug 13;13:4754. doi: 10.1038/s41467-022-32378-x (PMC9376099; doi:10.1038/s41467-022-32378-x)
Supplement: Supplementary file 3 — Description of Additional Supplementary Files [file 41467_2022_32378_MOESM3_ESM.pdf]

File name: Supplementary Movie 1

Description: Awake mouse. Three seconds of an awake mouse in recording both.

File name: Supplementary Movie 2

Description: Average visual evoked fast wave. Average of single trial LFP filtered at fast, 30-50Hz, frequencies over the cortical surface during the first 100 ms of the visual evoked response. The flash occurs at time = 0 ms. Note that the visual evoked gamma activity begins caudally within V1 and propagates rostrally.

File name: Supplementary Movie 3

Description: Average visual evoked slow wave. Average of single trial LFP filtered at slow, 3-6Hz, frequencies, over the cortical surface during the first 600 ms of the visual evoked response. The flash occurs at time = 0 ms. Note that the visual evoked gamma activity begins rostral to V1 in higher order cortical areas and propagates caudally.

File name: Supplementary Movie 4

Description: Both visual evoked waves superimposed. Superimposition of the average fast and slow filtered waves (amplitude of the signals is normalized to highlight temporal relationships between oscillations). The flash occurs at time = 0 ms. The fast wave begins caudal to the slow wave and travels anterolaterally towards the slow wave initiation zone.
