# Supplementary material for: Visual evoked feedforward–feedback traveling waves organize neural activity across the cortical hierarchy in mice
Source: Nat Commun. 2022 Aug 13;13:4754. doi: 10.1038/s41467-022-32378-x (PMC9376099; doi:10.1038/s41467-022-32378-x)
Supplement: Supplementary file 8 — Reporting Summary [file 41467_2022_32378_MOESM8_ESM.pdf]

nature portfolio

Corresponding author(s): Alex Proekt

Last updated by author(s): May 20, 2022

Reporting Summary

Nature Portfolio wishes to improve the reproducibility of the work that we publish. This form provides structure for consistency and transparency in reporting. For further information on Nature Portfolio policies, see our Editorial Policies and the Editorial Policy Checklist.

Statistics

For all statistical analyses, confirm that the following items are present in the figure legend, table legend, main text, or Methods section.

n/a	Confirmed	
☐	☒	The exact sample size (*n*) for each experimental group/condition, given as a discrete number and unit of measurement
☐	☒	A statement on whether measurements were taken from distinct samples or whether the same sample was measured repeatedly
☐	☒	The statistical test(s) used AND whether they are one- or two-sided<br>*Only common tests should be described solely by name; describe more complex techniques in the Methods section.*
☐	☒	A description of all covariates tested
☐	☒	A description of any assumptions or corrections, such as tests of normality and adjustment for multiple comparisons
☐	☒	A full description of the statistical parameters including central tendency (e.g. means) or other basic estimates (e.g. regression coefficient) AND variation (e.g. standard deviation) or associated estimates of uncertainty (e.g. confidence intervals)
☐	☒	For null hypothesis testing, the test statistic (e.g. *F*, *t*, *r*) with confidence intervals, effect sizes, degrees of freedom and *P* value noted<br>*Give P values as exact values whenever suitable.*
☒	☐	For Bayesian analysis, information on the choice of priors and Markov chain Monte Carlo settings
☐	☒	For hierarchical and complex designs, identification of the appropriate level for tests and full reporting of outcomes
☒	☐	Estimates of effect sizes (e.g. Cohen's *d*, Pearson's *r*), indicating how they were calculated

Our web collection on statistics for biologists contains articles on many of the points above.

Software and code

Policy information about availability of computer code

Data collection	The data in this study was collected using an Omniplex acquisition system and software (Plexon, version 18.3), and converted into .mat files using Plexon build SDKs (https://plexon.com/software-downloads/#software-downloads-SDKs, Version 1.8.3, downloaded March 2019).
Data analysis	All single units were identified using Kilosort (version: ,Kilosort 1.0, downloaded on 2/28/2020, available: https://github.com/cortex-lab/KiloSort). All analysis was performed using custom built Matlab (Mathworks, Matlab 2019a) code unless otherwise stated. Custom built code can be found https://doi.org/10.5281/zenodo.6578571.

For manuscripts utilizing custom algorithms or software that are central to the research but not yet described in published literature, software must be made available to editors and reviewers. We strongly encourage code deposition in a community repository (e.g. GitHub). See the Nature Portfolio guidelines for submitting code & software for further information.

Data

Policy information about availability of data

All manuscripts must include a data availability statement. This statement should provide the following information, where applicable:
- Accession codes, unique identifiers, or web links for publicly available datasets
- A description of any restrictions on data availability
- For clinical datasets or third party data, please ensure that the statement adheres to our policy

The raw data presented in this manuscript is available at https://doi.org/10.5281/zenodo.6578571.

Field-specific reporting

Please select the one below that is the best fit for your research. If you are not sure, read the appropriate sections before making your selection.

☒ Life sciences ☐ Behavioural & social sciences ☐ Ecological, evolutionary & environmental sciences

For a reference copy of the document with all sections, see nature.com/documents/nr-reporting-summary-flat.pdf

Life sciences study design

All studies must disclose on these points even when the disclosure is negative.

Sample size	We used approximately the same number of subjects as in previous work on this subject, such as Ferezou, I., Haiss, F., Gentet, L. J., Aronoff, R., Weber, B. & Petersen, C. C. H. Spatiotemporal Dynamics of Cortical Sensorimotor Integration in Behaving Mice. Neuron 56, 907–923 (2007). Moreover, the structure of the data and analysis that we utilize are not very well suited for a standard power analysis on preliminary data.
Data exclusions	Inclusion criteria for mice included the following: 1) presence of visual-evoked potentials (as defined by the absolute value of the average LFP response exceeding 5 standard deviations of pre-stimulus data within 100 ms after stimulus presentation) 2) histological verification of depth recording sites. With this inclusion criteria, we present data from 13 out of 20 mice.
Replication	Each mouse that meet inclusion criteria requirements was treated as a replicate in this experiment. Thus 13 out of 20 mice met the inclusion criteria required and were treated as replicates.
Randomization	This study demonstrated the spatial propagation of visual evoked activity compared to time shuffled surrogates (this was done by creating surrogate trials picked from random time points in the recording to uncouple the stimulus start time and data). The surrogate trial start times were chosen from a random uniform distribution of time points within each epoch.
Blinding	Blinding was not relevant for this study since it is not a randomized control trial. Experimenters were blinded to the histological verification of electrode placements and therefore were blind to the histological location of electrodes while analyzing each electrodes signal to minimize bias.

Reporting for specific materials, systems and methods

We require information from authors about some types of materials, experimental systems and methods used in many studies. Here, indicate whether each material, system or method listed is relevant to your study. If you are not sure if a list item applies to your research, read the appropriate section before selecting a response.

Materials & experimental systems

n/a	Involved in the study	
☒	☐	Antibodies
☒	☐	Eukaryotic cell lines
☒	☐	Palaeontology and archaeology
☐	☒	Animals and other organisms
☒	☐	Human research participants
☒	☐	Clinical data
☒	☐	Dual use research of concern

Methods

n/a	Involved in the study	
☒	☐	ChIP-seq
☒	☐	Flow cytometry
☒	☐	MRI-based neuroimaging

Animals and other organisms

Policy information about studies involving animals; ARRIVE guidelines recommended for reporting animal research

Laboratory animals	All experiments were performed using 8 male and 6 female adult (12– 32 weeks old, 20–30 g) C57BL/6 mice (Jackson Laboratories).Mice were housed under a reverse 12:12 h, light: dark cycle, with a temperature range of 73-77 degrees Fahrenheit and 30-70% humidity, and were provided with food and water ad libitum.
Wild animals	No wild animals were used
Field-collected samples	No field samples were collected
Ethics oversight	Institutional Animal Care and Use Committee at the University of Pennsylvania approved protocols required to complete this study.

Note that full information on the approval of the study protocol must also be provided in the manuscript.
